# Cognitive biases in shoulder surgery decision-making: a narrative review of hidden influences and mitigation strategies

**DOI:** 10.1016/j.jseint.2026.101731

**Published:** 2026-05-04

**Authors:** Christopher Fernainy, Matthew Frederickson, Mariano E. Menendez

**Affiliations:** a University of Nevada Reno School of Medicine, Reno, NV, USA; b Department of Orthopaedic Surgery, University of California Davis School of Medicine, Davis, CA, USA

**Keywords:** Cognitive bias, Orthopaedic surgery, Shoulder surgery, Bias mitigation, Decision making, Surgical judgment

## Abstract

Despite advances in evidence-based guidelines, three-dimensional pre-operative planning, and digital decision support, decision-making in shoulder surgery remains influenced by cognitive biases. These systematic errors in judgment, which arise from reliance on mental shortcuts, can alter how surgeons interpret information, select treatments, and evaluate outcomes. This review examines 7 biases commonly encountered in clinical practice—anchoring, availability, confirmation, sunk cost fallacy, outcome bias, commission bias, and overconfidence—and describes their potential to influence management decisions. Drawing from behavioral science and clinical experience, we illustrate the mechanisms by which these biases operate, how they may interact, and the implications for patient care, surgical training, and institutional practice. We propose strategies to recognize and reduce their impact, with the goal of promoting more unbiased, evidence-based, patient-centered decision-making.

Shoulder surgery has entered an era of unprecedented technical precision. With pre-operative three-dimensional planning, intraoperative navigation, patient-specific implants, and standardized clinical practice guidelines, one might expect surgical decisions to be increasingly consistent and evidence-driven. Nevertheless, significant variation remains in the timing of surgery, the choice of procedure, and its execution, even among the most experienced clinicians.

These differences are not fully explained by patient characteristics or institutional constraints. Research in cognitive psychology and behavioral economics has shown that decision-making is also shaped by cognitive biases, which are predictable patterns of deviation from rational judgment that often operate below the level of conscious awareness.^1^^,^^5^ Although well described in other medical fields, these biases are infrequently discussed in orthopedic surgery. They may be mistaken for clinical intuition or experience, blurring the distinction between expertise and error.

This review provides a framework for understanding common cognitive biases that influence decision-making in shoulder surgery, which can be applied to any subspecialty within orthopedic surgery. Using clinical examples, we illustrate how these biases can distort reasoning and affect patient care. We describe how high-pressure environments, time constraints, and financial or institutional factors may intensify bias. Finally, we outline strategies to help surgeons recognize and address these cognitive tendencies, with the aim of promoting more deliberate and patient-centered care. We would like to mention that as a narrative review, this work is inherently subject to selection and interpretive bias and does not quantify the relative prevalence or impact of individual cognitive biases in surgical practice.

## Anchoring bias: the power of first impressions

Anchoring occurs when a clinician places disproportionate weight on an initial piece of information and builds their entire thought process around it.^6^ This often happens unconsciously and can distort clinical judgment. For example, a patient may present with minimal pain and good shoulder function, but with an magnetic resonance imaging (MRI) that shows a full-thickness supraspinatus tear. If the surgeon has seen the image prior to the visit, they may unconsciously build the entire decision-making process around the tear, despite a more benign clinical picture. In another case, a referring physician describes the patient as having “clear recurrent instability,” anchoring the surgeon to that diagnosis before a physical exam or the patient has been asked a single question.

What makes anchoring particularly powerful is its visual or emotional salience. The data in an MRI carries implicit authority, often bypassing reflective reasoning. Even the way a radiology report is worded can serve as a cognitive anchor. This is compounded when images are viewed in high-stakes settings such as multidisciplinary meetings, where group consensus may form prematurely before a detailed case review.

One way to mitigate anchoring is to delay imaging review until after a thorough history and physical examination. Building the clinical narrative first, independent of any radiologic findings, can offer a more balanced perspective and reduce premature closure. Another technique is to generate at least 2 alternative diagnoses before viewing imaging, ensuring that the mind is primed to consider more than one explanatory pathway.

An additional and often underrecognized extension of anchoring bias originates from the patient. Patients who have already read imaging reports prior to attending follow-up appointments may already have formed preconceived expectations regarding their diagnosis and possibly surgical intervention. This can be driven by outside sources of information, such as internet searches or artificial intelligence applications. Information from these sources, paired with the imaging report, can cause anchoring bias for the patient regarding the injury and treatment options. Situations such as this also add an additional factor to the surgeon's own anchoring bias. The surgeon now needs to overcome not only their own bias, but the patient's. We recognize this is an area for potential improvement in patient care. In clinic visits, before imaging has been taken, physicians can emphasize history and physical examination findings before imaging. In addition, surgeons can educate the patient on potential imaging findings, ensuring that they understand evidence of certain pathology may not necessitate surgery.

## Availability bias: when the last case clouds the next

Availability bias refers to the tendency to overestimate the likelihood of events based on how easily examples come to mind.^7^ This is especially prevalent when a prior case had a dramatic or emotional outcome. Imagine a surgeon who recently treated a failed anatomic total shoulder arthroplasty that required revision. Even if this experience only happened once in a career, it may remain emotionally charged and cognitively accessible. In future patients with borderline cuff function, that memory nudges the surgeon toward reverse arthroplasty, even if the evidence base does not necessarily support that choice for the current patient.

Similarly, a complication during a Latarjet procedure, such as a graft fracture or post-operative infection, may lead a surgeon to avoid the procedure altogether. The issue is not whether the prior complication was real; it is whether that memory is being over-weighted in a way that biases current decisions. Interestingly, availability bias can also operate in reverse: a run of excellent results with a risky procedure may lead a surgeon to underestimate potential harm, a phenomenon sometimes described as the “streak effect.”^3^

To counteract availability bias, surgeons should reflect on whether their instincts are shaped by recent memory or reliable data. Seeking input from colleagues or briefly reviewing comparative studies can recalibrate one's perception of risk. Keeping a personal “outcome log” that includes both complications and uneventful recoveries can help contextualize isolated events against the broader spectrum of results.

## Confirmation bias: seeing only what we expect to see

Once a diagnostic or therapeutic hypothesis is formed, clinicians tend to seek out data that supports it while downplaying or ignoring disconfirming information. This is known as confirmation bias, and it is both common and difficult to detect.^4^ In shoulder surgery, an example of this might be interpreting equivocal clinical signs as consistent with rotator cuff pathology simply because the surgeon is already considering a reverse total shoulder arthroplasty. A weak belly-press test becomes “subscapular dysfunction,” even in the absence of objective imaging or a convincing history.

Radiology reports can also feed into this bias.^13^ If a clinician is already leaning toward a diagnosis of labral tear, a vague mention of “superior labral irregularity” may be taken as proof, even if that same phrase might have been dismissed in another context. Confirmation bias can even influence intraoperative decision-making, such as interpreting borderline cartilage wear as significant to justify an arthroplasty that was already favored pre-operatively.

The key to mitigating confirmation bias lies in deliberately seeking out evidence that challenges one's assumptions.^9^ Asking oneself, “what would I expect to find if my hypothesis were incorrect?” can open alternative possibilities. Consulting a colleague who may approach the case from a different angle can also disrupt this self-reinforcing loop. Some surgeons find it helpful to assign a “devil's advocate” in pre-operative conferences whose explicit role is to argue against the prevailing plan, forcing the team to re-examine their assumptions.

## Sunk cost fallacy: when past investment clouds present judgment

The sunk cost fallacy refers to the reluctance to abandon a course of action due to prior investment in time, effort, or emotion.^2^ In surgery, this often manifests as persistent attempts to make a strategy work even when evidence suggests a change is needed. Consider the case of a young patient who has undergone 2 failed arthroscopic Bankart repairs. Rather than recommending a glenoid bone reconstruction procedure, the surgeon offers a third arthroscopic repair, convinced that with a few technical adjustments, success is still possible.

Intraoperative examples are equally common. A surgeon placing a glenoid baseplate may recognize suboptimal positioning during trialing. Yet instead of revising the preparation, they proceed, rationalizing that “it is close enough.” The motivation is rarely laziness, but rather an emotional investment in avoiding the acknowledgment that something must be undone and redone. Even in research, sunk costs can manifest, such as continuing to pursue a flawed hypothesis because of the time already spent collecting preliminary data.

Overcoming the sunk cost fallacy requires emotional flexibility. Recognizing that changing course is not a sign of failure, but rather an act of professional maturity, can reduce the stigma associated with revision or replanning. Case discussions and morbidity and mortality meetings are useful forums for normalizing such course corrections. Institutions can further help by fostering nonpunitive environments where mid-course changes are celebrated as adaptive expertise rather than criticized as indecision.

In addition, the sunk cost fallacy may be amplified by systemic and cultural factors within modern surgical practice. Time pressures for operative room efficiency, productivity expectations, and payment models that are based on relative value units may influence the actions of surgeons. These external factors may discourage surgeons from redoing steps to make intraoperative course corrections in an effort to save time. There is also the pressure and expectations of the operative staff, further influencing surgeons to not to undo steps of a procedure if a correction is needed. This highlights the fact that cognitive biases are not purely individualized; rather, they are reinforced by the environment in which the surgeon practices. However, this is not to say that creating a surgical environment where final outcomes are prioritized is impossible; the biases need to be addressed and the environment reshaped.

## Outcome bias: mistaking results for reasoning

Outcome bias occurs when decisions are judged based on the eventual result rather than the quality of the decision-making process.^1^^,^^11^ In shoulder surgery, this bias is pervasive and subtly shapes both individual learning and institutional culture. A favorable result may reinforce a decision, or future decisions, regardless of whether it was evidence-based. Conversely, an unfavorable outcome may lead to criticism of an otherwise appropriate and well-reasoned approach. In this way, outcome bias can influence how decisions are interpreted after the fact, creating a feedback loop in which results, rather than reasoning, shape future clinical judgment.

This bias undermines reflective practice by creating a feedback loop where good outcomes validate any process and bad outcomes cast doubt on even the best-intentioned strategies. It can also lead to “outcome-driven learning,” where rare flukes are internalized as reliable patterns.

A culture of reviewing not just complications but also “good” outcomes can help break this loop. A systematic review by Aylmore et al^1^ identified that reflecting on the reasoning behind clinical decisions can help reduce surgical errors. This can be applied through open discussion where instead of asking only, “What went wrong?” after failure, we should also ask, “Did we get this right for the right reasons?” when things go well. Structured after-action reviews that de-emphasize the eventual outcome and focus on decision quality can help embed this discipline.

## Commission bias: the pressure to act

Surgeons are trained to act. In many ways, the surgical mindset is defined by intervention. Commission bias refers to the impulse to do something—anything—rather than take a watchful or conservative approach.^8^ Sometimes this can be an appropriate course of action, but it becomes problematic when action is chosen primarily to relieve uncertainty or satisfy expectations.

In the world of shoulder surgery, this bias might lead to recommending total shoulder arthroplasty for patients with mild osteoarthritis who have not completed a full course of conservative nonoperative management. It might also drive labral repair surgery in athletes with minimal symptoms, based more on the surgeon's anxiety than the patient's risk profile. In extreme cases, commission bias can push surgeons toward simultaneous bilateral procedures or unnecessary concomitant interventions during unrelated surgeries.

Commission bias is reinforced by structural and cultural factors: productivity metrics, patient expectations, and the prestige of surgery.^8^ The antidote is shared decision-making. When patients are given clear, balanced information about risks and benefits, many opt for conservative management, and their choice can temper the surgeon's bias to intervene. Another helpful tool is the “consider doing nothing” checklist—an intentional pause before surgical scheduling where the team discusses whether inaction might be the most evidence-aligned choice.^10^

Structural factors within modern health care systems may further reinforce commission bias. Reimbursement and productivity models can incentivize procedural interventions and higher-complexity decision-making, while nonoperative management may be perceived as less productive despite being clinically appropriate. Strategies to mitigate this include emphasizing shared decision-making, incorporating peer-to-peer discussion in borderline cases, and utilizing structured or algorithmic approaches to clinical decision-making that consistently reinforce consideration of nonoperative management when appropriate.

## Overconfidence bias: immunity to bias is its own bias

Among the most insidious biases is the belief that we are not biased. Overconfidence bias leads us to assume that experience and skill make us more rational, more objective, or less fallible than others.^1^^,^^12^ This mindset is common among highly trained surgeons and can be reinforced by good outcomes or professional accolades.

This bias manifests as resistance to peer feedback, dismissal of divergent opinions, and blind spots in self-assessment. It is especially dangerous because it undermines the very practices that mitigate other biases, such as reflection, open-mindedness, and humility. It may also contribute to “diagnostic momentum,” in which a senior surgeon's early opinion shapes the entire team's direction, discouraging dissent from trainees or colleagues.

Cultivating intellectual humility is key. Even experienced surgeons benefit from case discussions, audit data, and the occasional challenge from a younger colleague. The most seasoned clinicians are often those most aware of their own blind spots. Embedding peer review into routine practice, not just for complications but for all complex cases, can normalize the idea that every surgeon's thinking is worth examining, regardless of seniority.

## Toward a more reflective surgical culture

Cognitive biases are not signs of incompetence. They are the natural byproducts of being human and being a surgeon. The goal is not to eliminate them but to make them identifiable and therefore manageable. Importantly, biases rarely occur in isolation; a single case may involve anchoring on an MRI finding, reinforced by confirmation bias from selective literature reading, and sealed by overconfidence bias in dismissing contrary advice. Recognizing these groupings can be particularly valuable.

In addition to individual cognitive processes, external influences such as industry relationships and emerging technologies may further shape clinical decision making. These factors represent additional sources of both cognitive and systemic bias that warrant further evaluation.

Creating systems that support reflective decision-making is essential. This includes structured pre-operative planning meetings, routine outcome audits, use of patient decision aids, and multidisciplinary discussions of complex or borderline cases. Incorporating simulation-based training with deliberate exposure to bias-prone scenarios can also help surgeons recognize these patterns in real time. Embedding these strategies into the culture of shoulder surgery can promote more informed, individualized, and unbiased care.

From a medicolegal perspective, awareness of cognitive bias is increasingly important. In litigation, the quality of the decision-making process is scrutinized as much as the outcome. Demonstrating that decisions were made through a structured, evidence-based process can be a powerful defense.

## Conclusion

Ultimately, acknowledging that susceptibility to cognitive bias affects even the most experienced surgeons is not a sign of weakness, but rather a step toward greater wisdom. By integrating awareness of bias into both individual practice and institutional systems, shoulder surgeons can align closer to the twin goals of science and humanity: making the best possible decision for every patient, every time.

## Disclaimers:

Funding: No funding was disclosed by the authors.

Conflicts of interest: Mariano E. Menendez, MD serves as a consultant for Stryker. Any additional authors, their immediate families, and any research foundations with which they are affiliated have not received any financial payments or other benefits from any commercial entity related to the subject of this article.
